# Prognostic significance and risk factors for pelvic and para-aortic lymph node metastasis in type I and type II ovarian cancer: a large population-based database analysis

**DOI:** 10.1186/s13048-023-01102-8

**Published:** 2023-01-30

**Authors:** Hailin Yu, Jieyu Wang, Beibei Wu, Jun li, Ruifang Chen

**Affiliations:** Obstetrics and Gynecology Hospital, Fudan University, Shanghai, China

**Keywords:** Ovarian cancer, Two-tier classification system, Lymph node metastasis, Para-aortic lymph nodes, Pelvic lymph nodes, Prognosis, Risk factors

## Abstract

**Background:**

To compare the prognosis of lymphatic metastasis in type I and type II epithelial ovarian cancer (OC) and to identify the risk factors for pelvic lymph node metastases (PLNs) and para-aortic lymph node metastases (PALNs).

**Methods:**

Patients diagnosed with epithelial OC were collected from the Surveillance, Epidemiology, and End Results (SEER) database. Overall survival (OS) and cancer-specific survival (CSS) were estimated. The Cox proportional hazards regression model was used to identify independent predictors of survival.

**Results:**

A total of 11,275 patients with OC were enrolled, including 31.2% with type I and 68.8% with type II. Type II and high tumour stage were risk factors for lymph node involvement (*p* < 0.05). The overall rate of lymph node metastasis in type I was 11.8%, and that in type II was 36.7%. In the type I group, the lymph node metastasis rates in stages T1, T2, T3 and T_X_M_1_ were 3.2%, 14.5%, 40.4% and 50.0%, respectively. In the type II group, these rates were 6.4%, 20.4%, 54.1% and 61.1%, respectively. Age and tumour size had little effect on lymph node metastasis, and grade 3 was not always a risk factor. For the type I group, the 10-year CSS rates of LN(-), PLN( +), PALN( +), and PLN + PALN( +) were 80.6%, 46.6%, 36.3%, and 32.3%, respectively. The prognosis of PLN ( +) was better than that of PALN ( +) in the type I group (*p* > 0.05). For the type II group, the 10-year CSS rates of LN(-), PLN( +), PALN( +), and PLN + PALN( +) were 55.6%, 18.5%, 25.7%, and 18.2%, respectively. PALN ( +) had a significantly better prognosis than PLN ( +) in the type II group (*p* < 0.05).

**Conclusions:**

The clinical characteristics and prognoses of patients with type I and type II OC differed greatly. Patients with type II and higher tumour stages had poorer prognoses. Type I with PALN metastasis and type II with PLN metastasis indicated a worse prognosis. Patients with stage TI did not require lymph node dissection, especially in the type I group.

## Background

Ovarian cancer (OC) has the most unfavourable prognosis among gynaecological cancers. With the rapid development of molecular genetics and clinical pathology, the two-tier grading system for ovarian cancer has been widely recognized and accepted. This theory was first proposed in 2004 [1] and divides epithelial ovarian cancer into two types, type I and type II. Type I OC tends to consist of low-grade tumours that arise from borderline disease, whereas type II tumours are high-grade neoplasms without morphologically recognizable precursor lesions. For serous tumours, low-grade tumours are type I tumours, and high-grade tumours are type II tumours. In addition to low-grade serous disease, type I tumours are composed of the mucinous type, the low-grade endometrioid type, malignant Brenner tumours and clear cell carcinomas. Type II tumours include high-grade serous carcinomas, the high-grade endometrioid type, carcinosarcoma and undifferentiated carcinoma. Regarding distinct molecular changes, type I tumours are associated with *BRAF*, *KRAS*, *CTNNB1* and *PTEN* mutations, as well as microsatellite instability. However, the p53 mutation appears frequently in type II tumours. This model of carcinogenesis provides a morphological and molecular framework for studies aimed at elucidating the pathogenesis of ovarian cancer.

In the majority of cases, the primary routes of OC metastasis are through four different means: direct spread, implantation on distant intra-abdominal sites, lymphogenous and haematogenous. Concerning lymphatic spread, para-aortic lymph nodes and pelvic lymph nodes [2–4] are the common sites involved and are the most important prognostic factors in ovarian cancer. It is commonly believed that the para-aortic lymph node is the first site of lymphatic spread for the ovary, followed by the pelvic lymph node. Thus, lymphadenectomy, including both pelvic and para-aortic lymph nodes, is currently the standard primary therapy for OC. However, it was reported that the incidence of complications with lymph node adenectomy was approximately 5.9% to 24%, including immediate complications, including vascular injury, lymphocyte cyst, small intestinal obstruction, deep vein thrombosis, urinary fistula, postoperative infection, etc. [5, 6]; and long-term complications, including lower extremity lymphedema, sometimes seriously affecting the patient's quality of life. Moreover, lymph nodes participate in the immune response, and lack of function causes disconnection of tumour immune circulation and failure of immune supervision. The side effects of lymphadenectomy are significantly increased, especially for para-aortic lymph node dissection [7–9]. A recent Lion clinical trial [7–9] suggested that systematic pelvic and para-aortic lymphadenectomy is not recommended in patients with advanced ovarian cancer (International Federation of Gynecology and Obstetrics (FIGO) Stage IIIB to IV) who had undergone intra-abdominal macroscopically complete resection and had not developed enlarged lymph nodes either before or during surgery. Thus, systematic lymphadenectomy is not always necessarily performed in all patients, and the benefit of lymphadenectomy remains ambiguous [11, 12].

Identifying differences between type I and type II OC patients and the underlying risk factors for lymph node metastases would aid in the proper determination of lymphadenectomy and allow for the most accurate planning of adjuvant treatment. Furthermore, it makes sense to analyse the prognostic significance and different risk factors for pelvic and para-aortic lymph node metastasis in patients with type I and type II ovarian cancer. It was reported that age, histology, grade, and CA125 level influenced the incidence of nodal metastasis. However, due to the limitations and small sample sizes of prior studies, little information exists about the lymph node status of patients with type I or type II disease [13–15]. Therefore, further studies are needed to explore the differences in lymph node status in OC patients with different types. Thus, we performed a large population-based database analysis to evaluate the prognostic significance and risk factors for pelvic and para-aortic lymphatic spread in patients with type I and type II ovarian cancer.

## Methods

**Fig. 1:**
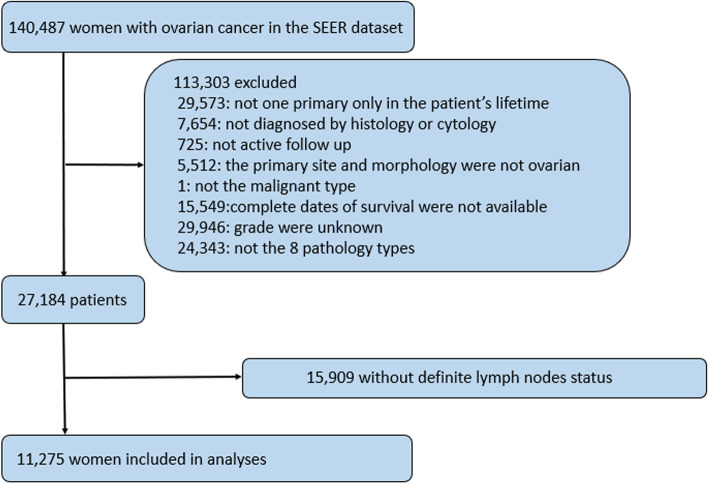
The flow diagram of participants recruited

Demographic, clinicopathologic, and survival information were generated from the Surveillance, Epidemiology, and End Results (SEER) database (http://seer.cancer.gov/). All surgically treated primary OC patients were identified from January 1, 1988, to December 31, 2013. The following were the inclusion criteria: (1) lymph node status available; (2) only one primary cancer in the patient’s lifetime; (3) survival time available, and (4) clear grade. A total of 11,275 patients were finally obtained (Fig. 1). Collected data included each patient's age, tumour size, tumour grade and type, FIGO stage, and nodal status (number of pelvic and aortic lymph nodes removed, number of metastatic lymph nodes and distribution of metastatic lymph nodes). To determine type I or type II disease, we classified the ICD-O-3 codes (8380, 8460 and 8461) by grade. Grades 1 and 2 were defined as low grade, which belonged to type I disease. At the same time, grade 3 was defined as high grade and belonged to type II disease. In addition, type I also included clear cell adenocarcinoma (ICD-O-3: 8310) and mucinous adenocarcinoma (ICD-O-3: 8480). Type II included undifferentiated carcinoma (ICD-O-3: 8020) and carcinosarcoma (ICD-O-3: 8980). The work has been reported in line with the STROCSS criteria [16].

Statistical analysis was performed using chi-square (χ^2^) analysis. Survival was estimated by the Kaplan–Meier method and assessed by the log-rank test. To investigate the risk factors for pelvic and para-aortic lymph node metastasis, multivariate analysis was performed using the logistic proportional hazards model in different types. Analyses were performed using the SPSS statistical software package, version 19.0 (IBM Corporation, Armonk, NY, USA). All tests were two-tailed, and statistical significance remained conventionally defined as *p* < 0.05 in all other cases.

## Results

### Patient characteristics in type I and type II OC

A total of 11,275 patients with OC were enrolled in our study, including 31.2% with type I (*n* = 3522) and 68.8% with type II (*n* = 7753). The median patient age was 56.0 years old (range: 15–96), and 71.8% of patients were ≥ 50 years old. For patients in different age groups (< 50 y vs. ≥ 50 y), lymph node metastasis was seen at a similar rate (24.4% vs. 30.7%). In the two-tier grading system, the proportion of different tumour stages varied greatly. Type I was dominated by stage T1 (68.1%), and type II was dominated by stage T3 (52.3%). Patients with type II had an obviously higher tendency towards lymph metastasis than those with type I (36.7% vs. 11.8%). The higher that the tumour stage was in Stages T1, T2, T3 and T_X_M1, the more patients that showed metastasis in both the type I group (3.2%, 14.5%, 40.4%, 50.0%) and the type II group (6.4%, 20.4%, 54.1%, 61.1%). However, 4360 patients in stage T1 in our study had an extremely low rate (4.6%) of lymph node metastasis, especially those in the type I group with 1.6% PLN( +), 1.3% PALN( +) and 0.3% PLN + PALN( +). For the traditional grading system, the positive rates of PLN (5.1%, 9.2%, 17.2%), PALN (2.9%, 7.1%, 15.5%) and PLN + PALN (1.1%, 2.7%, 5.9%) were higher accordingly from grades 1 to 3. Except for 27.2% of patients with unknown tumour sizes, the proportions of patients with tumour sizes ≤ 5 cm, ≤ 15 cm and > 15 cm were 15.9%, 42.1% and 14.8%, respectively. Tumour size > 15 cm was more common in patients with type I (23.2%) than type II disease (10.9%). Contrary to the increased tumour size, the rate of lymph node metastasis decreased from 28.4% to 28.3% to 19.7% (Tables 1, 2 and 3).

**Table 1:** Clinicopathological characteristics of type I and type II ovarian cancer

	**All patients**		**Type I**		**Type II**	
	**n**	**Percent**	**n**	**Percent**	**n**	**Percent**
**Total**	11,275	NA	3522	31.2%	7753	68.8%
**Age (year)**						
< 50	3174	28.2%	1376	39.1%	1798	23.2%
≥ 50	8101	71.8%	2146	60.9%	5955	76.8%
**Tumor Stage**						
Stage T1	4360	38.7%	2397	68.1%	1963	25.3%
Stage T2	1727	15.3%	441	12.5%	1286	16.6%
Stage T3	4658	41.3%	606	17.2%	4052	52.3%
Stage TxM1	482	4.3%	60	1.7%	422	5.4%
Unknown	48	0.4%	18	0.5%	30	0.4%
**Tumor size**						
≤ 5 cm	1795	15.9%	535	15.2%	1260	16.2%
5-10 cm	2553	22.6%	662	18.8%	1891	24.4%
10-15 cm	2200	19.5%	736	20.9%	1464	18.9%
15-20 cm	1034	9.2%	452	12.8%	582	7.5%
> 20 cm	628	5.6%	367	10.4%	261	3.4%
Unknown	3065	27.2%	770	21.9%	2295	29.6%

**Table 2:** Clinicopathological characteristics of ovarian cancer with and without lymph node metastasis

	**Negative** l**ymph node n (%)**	**Positive lymph node**								
		**Pelvic**			**Para-aortic**			**Pelvic + Para-aortic**		
		**n (%)**	**OR (95% CI)**	***p***	**n (%)**	**OR (95% CI)**	***p***	**n (%)**	**OR (95% CI)**	***p***
**Total**	8010 (71.0)	1495(13.3)	NA	NA	1285(11.4)	NA	NA	485 (4.3)	NA	NA
**Age (year)**										
< 50	2398 (75.6)	356 (11.2)	1		283 (8.9)	1		137 (4.3)	1	
≥ 50	5612 (69.3)	1139(14.0)	0.93(0.77–1.12)	0.447	1002(12.4)	0.89(0.77–1.04)	0.149	348 (4.3)	0.68(0.53–0.87)	**0.002**
**Histology**										
Type I	3105 (88.2)	205 (5.8)	1		167 (4.7)	1		45 (1.3)	1	
Type II	4905 (63.3)	1290(16.6)	1.32(1.06–1.65)	**0.015**	1118(14.4)	1.30(1.05–1.61)	**0.018**	440 (5.7)	1.85(1.29–2.65)	**0.001**
**Tumor Stage**										
Stage T1	4158 (95.4)	88 (2.0)	1		101 (2.3)	1		13 (0.3)	1	
Stage T2	1401 (81.1)	143 (8.3)	4.03(2.99–5.43)	**0.000**	149 (8.6)	3.78(2.85–5.01)	**0.000**	34 (2.0)	4.68(2.32–9.43)	**0.000**
Stage T3	2219 (47.6)	1134(24.3)	17.16(13.36–22.05)	**0.000**	916 (19.7)	11.31(8.92–14.36)	**0.000**	389 (8.4)	22.86(12.66–41.28)	**0.000**
Stage T_X_M1	194 (40.2)	127 (26.3)	23.15(16.30–32.89)	**0.000**	114 (23.7)	15.22(10.76–21.53)	**0.000**	47 (9.8)	30.39(15.08–61.23)	**0.000**
Unknown	38 (79.2)	3 (6.2)			5 (10.4)			2 (4.2)		
**Pathology Grade**										
Grade 1	1534 (90.9)	85 (5.1)	1		49 (2.9)	1		19 (1.1)	1	
Grade 2	2423 (81.0)	276 (9.2)	0.86(0.61–1.22)	0.404	212 (7.1)	1.35(0.91–1.98)	0.134	80 (2.7)	0.86(0.61–1.22)	0.404
Grade 3	4053 (61.4)	1134(17.2)	1.18(0.85–1.64)	0.316	1024 (15.5)	2.22(1.55–3.20)	**0.000**	386 (5.9)	1.18(0.85–1.64)	0.316
**Tumor size**										
≤ 5 cm	1285 (71.6)	235 (13.1)	1		181 (10.1)	1		94 (5.2)	1	
5-10 cm	1767 (69.2)	376 (14.7)	1.02(0.86–1.21)	0.820	284 (11.1)	0.99(0.83–1.18)	0.881	126 (5.0)	0.88(0.67–1.17)	0.391
10-15 cm	1643 (74.7)	229 (10.4)	0.85(0.71–1.02)	0.081	242 (11.0)	1.16(0.96–1.40)	0.132	86 (3.9)	0.86(0.63–1.17)	0.337
15-20 cm	814 (78.7)	98 (9.5)	0.82(0.64–1.05)	0.110	99 (9.6)	1.06(0.83–1.36)	0.642	23 (2.2)	0.58(0.36–0.93)	**0.023**
> 20 cm	520 (82.8)	36 (5.7)	0.61(0.43–0.86)	**0.005**	61 (9.7)	1.36(1.00–1.85)	**0.049**	11 (1.8)	0.59(0.31–1.13)	0.113
Unknown	1981 (64.6)	521 (17.0)			418 (13.7)			145 (4.7)

**Table 3:** PLN and PALN metastasis in different tumor stage of type I and type II OC

Tumor Stage	lymph node in type I n (%)		lymph node in type II n (%)		PLN( +) n (%)		PALN( +) n (%)		PLN( +) + PALN( +) n (%)	
	**Negative**	**Positive**	**Negative**	**Positive**	**Type I**	**Type II**	**Type I**	**Type II**	**Type I**	**Type II**
**Stage T1**	2320(96.8)	77(3.2)	1838 (93.6)	125(6.4)	38(1.6)	50 (2.6)	32(1.3)	69 (3.5)	7(0.3)	6 (0.3)
**Stage T2**	377(85.5)	64(14.5)	1024 (79.6)	262(20.4)	28(6.4)	115 (8.9)	30(6.8)	119 (9.3)	6(1.4)	28 (2.2)
**Stage T3**	361(59.6)	245(40.4)	1858 (45.9)	2194(54.1)	124(20.5)	1010 (24.9)	92(15.2)	824 (20.3)	29(4.8)	360 (8.9)
**Stage TxM1**	30(50.0)	30(50.0)	164 (38.9)	258(61.1)	15(25.0)	112 (26.5)	12(20.0)	102 (24.2)	3(5.0)	44 (10.4)
**Unknown**	17(94.4)	1(5.6)	21 (70.0)	9(30.0)	0(0.0)	3 (10.0)	1(5.6)	4 (13.3)	0(0.0)	2 (6.7)

### Analysis of the risk factors for PLN and PALN metastasis in type I and type II OC

To explore whether different risk factors exist in type I and type II, we divided the patients into 2 groups and analysed the risk factors (Tables 3 and 4). The tumour staging system was the only risk factor with statistical significance in all six groups (*p* < 0.001). The higher that the stage was from stage T1, T2, T3 to T_X_M1, the more likely that the patient was to have PLN involvement (type I: 1.6% vs. 6.4% vs. 20.5% vs. 25.0%, type II: 2.6% vs. 8.9% vs. 24.9% vs. 26.5%), PALN involvement (type I: 1.3% vs. 6.8% vs. 15.2% vs. 20.0%, type II: 3.5% vs. 9.3% vs. 20.3% vs. 24.2%) and PLN + PALN involvement (type I: 0.3% vs. 1.4% vs. 4.8% vs. 5.0%, type II: 0.3% vs. 2.2% vs. 8.9% vs. 10.4%) in both the type I and type II groups. In our study, age and tumour size had little effect on lymph node metastases. Patients aged ≥ 50 years old were less likely to have PLN + PALN metastasis in the type II group (OR = 0.63, *p* = 0.001). For tumour size, it was shown that tumour size > 15- ≤ 20 cm was related to less PLN metastasis (OR = 0.54, *p* = 0.006) and > 20 cm with less PLN + PALN metastasis (OR = 0.51, *p* = 0.013) in the type II group. In the traditional pathologic grading system, grade 3 was a risk factor for PLN( +) in type II patients (OR = 1.38, *p* < 0.001). It was also a risk factor for PALN( +) regardless of type classification (type I: OR = 2.28, *p* < 0.001; type II:OR = 1.66, *p* < 0.001).

**Table 4:** Multivariate analysis on PLN and PALN metastasis in type I and type II ovarian cancer

	**PLN**				**PALN**				**PLN + PALN**			
	**Type I**		**Type II**		**Type I**		**Type II**		**Type I**		**Type II**	
	**OR (95% CI)**	***p***	**OR (95% CI)**	***p***	**OR (95% CI)**	***p***	**OR (95% CI)**	***p***	**OR (95% CI)**	***p***	**OR (95% CI)**	***p***
**Age(yr)**												
˂50	1		1		1		1		1		1	
≥ 50	0.80(0.58–1.11)	0.181	0.85(0.72–1.01)	0.065	0.88(0.62–1.25)	0.474	0.90(0.75–1.06)	0.206	0.90 (0.45–1.76)	0.748	0.63(0.48–0.82)	**0.001**
**Tumor Stage**												
Stage T1	1		1		1		1		1		1	
Stage T2	4.40(2.59–7.47)	**0.000**	3.73(2.57–5.42)	**0.000**	5.26(3.18–9.05)	**0.000**	3.16(2.26–4.42)	**0.000**	3.79(1.07–13.51)	**0.040**	5.57(2.23–13.93)	**0.000**
Stage T3	19.54(13.17–29.16)	**0.000**	15.70(11.37–21.67)	**0.000**	15.14(9.93–23.10)	**0.000**	9.76(7.33–12.98)	**0.000**	18.16(7.40–44.53)	**0.000**	26.44(11.72–59.63)	**0.000**
Stage TxM1	33.04(14.96–72.93)	**0.000**	20.59(13.59–31.17)	**0.000**	23.92(10.34–55.37)	**0.000**	12.98(8.77–19.21)	**0.000**	21.11(4.09–108.88)	**0.000**	35.24(14.29–86.91)	**0.000**
**Pathology Grade**												
1	1				1				1			
2	0.84(0.50–1.38)	0.485	1		1.14(0.64–2.00)	0.662	1		0.77(0.25–2.37)	0.642	1	
3	1.12(0.79–1.58)	0.521	1.38(1.15–1.65)	**0.000**	2.28(1.57–3.33)	**0.000**	1.66(1.38–2.00)	**0.000**	1.24(0.61–2.51)	0.556	1.21(0.89–1.64)	0.232
**Tumor size**												
≤ 5 cm	1		1		1		1		1		1	
> 5- ≤ 10 cm	0.96(0.59–1.55)	0.858	1.03(0.86–1.23)	0.766	1.19(0.68–2.08)	0.538	0.96(0.80–1.16)	0.705	1.26(0.46–3.45)	0.651	0.86(0.64–1.15)	0.298
> 10- ≤ 15 cm	0.84(0.52–1.37)	0.495	0.85(0.70–1.04)	0.110	1.21(0.70–2.10)	0.494	1.14(0.94–1.40)	0.190	1.00(0.35–2.84)	0.998	0.85(0.61–1.17)	0.317
> 15- ≤ 20 cm	1.03(0.60–1.76)	0.912	0.76(0.57–1.00)	0.051	1.31(0.71–2.40)	0.388	1.01(0.76–1.33)	0.964	1.03(0.32–3.31)	0.959	0.51(0.30–0.87)	**0.013**
> 20 cm	0.74(0.40–1.39)	0.353	0.54(0.35–0.84)	**0.006**	1.80(0.97–3.37)	0.065	1.24(0.85–1.80)	0.266	0.69(0.17–2.86)	0.606	0.59(0.28–1.25)	0.171

### Comparison of survival rates of PLN and PALN metastasis in type I and type II OC

**Fig. 2:**
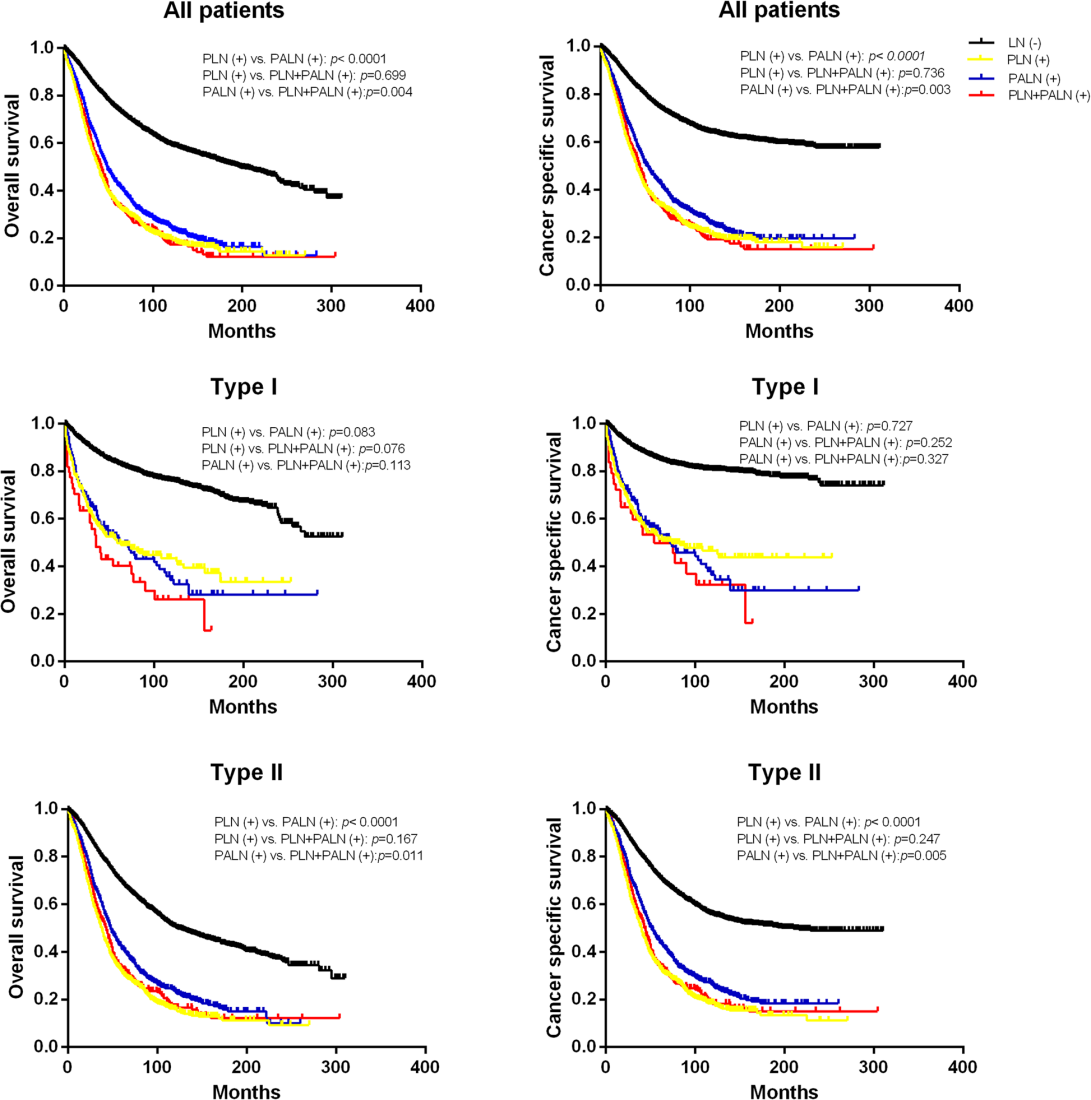
Overall survival and cancer-specific survival of patients. **A** Overall survival of all patients; **B** cancer-specific survival of all patients; **C** cancer-specific survival of type I patients; **D** overall survival of type I patients; **E** overall survival of type II patients; **F** cancer-specific survival of type II patients

In our study, the 10-y OS and 10-y CSS were 49.0% and 53.3%, respectively, in all patients with OC. However, the prognosis of patients in the type I group was much better than that of patients in the type II group (10-y OS 71.5% vs. 38.8%, 10-y CSS 75.9% vs. 43.0%). Compared with PLNs, PALNs are far from the primary site of ovarian cancer. However, among all patients, the prognosis of patients with PALN metastasis (10-y OS 24.6%, 10-y CSS 27.1%) was better than that of patients with PLN involvement (10-y OS 19.7%, 10-y CSS 22.4%) and PLN + PALN involvement (10-y OS 17.4%, 10-y CSS 19.5%) (Table 5, Fig. 2A and B). Because the pathogenesis of type I and type II is different, we explored the survival status of PLNs and PALNs in different types. In the type I group, PALN involvement (10-y OS 34.3%, 10-y CSS 36.3%) was associated with poorer OS and CSS than PLN involvement (10-y OS 43.5%, 10-y CSS 46.6%) (OS *p* = 0.083, CSS *p* = 0.727) but better survival than PLN + PALN involvement (10-y OS 26.1%, 10-y CSS 32.3%) (OS *p* = 0.113, CSS *p* = 0.327), although these differences were not significant (Fig. 2C and D). Nonetheless, in the type II group, patients with PALN metastasis had a more favourable prognosis (10-y OS 23.1%, 10-y CSS 25.7%) (OS: *p* < 0.0001; CSS: *p* < 0.0001). The 10-y OS and 10-y CSS were 15.9% and 18.5%, respectively, when PLN was involved and 16.5% and 18.2%, respectively, when PLN + PALN was involved in the type II group (Fig. 2E and F).

**Table 5:** The 10-y OS and 10-CSS of type I and II patients

	**All patients**			**Type** I			**Type** II		
	**Total N**	**10-y OS** **n(%)**	**10-y CSS** **n(%)**	**N**	**10-y OS** **n(%)**	**10-y CSS** **n(%)**	**N**	**10-y OS** **n(%)**	**10-y CSS** **n(%)**
**LN (-)**	8010	4832 (60.3)	5230 (65.3)	3105	2360 (76.0)	2503 (80.6)	4905	2472 (50.4)	2727 (55.6)
**PLN ( +)**	1495	294 (19.7)	334 (22.4)	205	89 (43.5)	96 (46.6)	1290	205 (15.9)	239 (18.5)
**PALN ( +)**	1285	316 (24.6)	348 (27.1)	167	57 (34.3)	61 (36.3)	1118	258 (23.1)	287 (25.7)
**PLN + PALN ( +)**	485	84 (17.4)	95 (19.5)	45	12 (26.1)	15 (32.3)	440	73 (16.5)	80 (18.2)
**Total**	11,275	5526 (49.0)	6007 (53.3)	3522	2518 (71.5)	2673 (75.9)	7753	3008 (38.8)	3333 (43.0)

## Discussion

In previous studies, the target for biopsy or lymphadenectomy largely depended on the size of the lymph nodes. However, size is not a reliable indicator of the status of nodal involvement [17, 18]. Currently, whether to perform lymph node dissection generally depends on clinicopathological data. Previous studies have identified multiple factors that affect the risk for lymph node metastasis. Factors shown to increase risk include grade, serous histology, CA125 level, bilateral primary lesion, positive cytologic washings, and ascites [19–21]. However, the amount of data in these studies was not sufficient. The prognostic impact of clinicopathological factors associated with node involvement should be investigated in larger studies to improve the prognostic relevance of node metastasis. The current study utilized data collected from the SEER database to explore the risk factors and prognostic value of lymph node metastasis and to differentiate these findings in a two-tier classification system.

### Risk factors for PLN and PALN metastasis in OC

In this series, our results showed that the occurrence of lymph node metastases is lower than that reported in the previous literature [11, 22, 23]. According to the traditional view, PALN metastasis is commonly understood as the initial route, with the pelvic nodes constituting a second metastatic site [23, 24]. However, in each tumour stage of the SEER data, the frequency of lymph node metastasis in the para-aortic basin is similar to that of the pelvic basin from tumour stage T1 to tumor stage TxM1. Through multivariate analysis, tumour stage was confirmed as an independent risk factor for node involvement in our study, in accordance with the data in previous studies [25]. The higher that the tumour stage is, the greater that the chance is of lymph node metastasis. Thus, considering the extremely low rate of positive lymph nodes at tumour stage T1, the benefits of lymphadenectomy should be weighed in this group, especially in the type I group. In the higher tumour stage T2-TxM1, lymph nodes should be screened throughout the pelvic and para-aortic regions to maximize the chance of finding positive lymph nodes.

There is accumulating evidence that G3 is a risk factor for lymph node metastasis [20, 26, 27]. However, the pathological grading system was not always a risk factor in this analysis, in contrast to the data in the literature [19, 26]. Thus, utilizing the pathological grade system retains limited function in the ability to detect those at risk for nodal disease. New classifications should be explored.

### PLN and PALN metastasis in type I and type II OC

According to histologic pathogenesis, molecular alterations, and clinicopathologic features, the classification of ovarian cancers includes two distinct subtypes. Whether from a clinical perspective or molecular alterations, type I is different from type II [28–34]. Based on these different aspects, the two types should have stratified treatment plans. However, there is currently no research that differentiates the role of lymph node metastasis between the two subtypes. The uniqueness of the current study lies in its stratification of patients not only by traditional pathologic factors but also by the two-tier system. In our cohort, the incidences of PLN, PALN and PLN + PALN metastasis in type II patients were almost threefold higher than those in type I patients. In multivariate analysis, compared to type I, type II was a significant and independent risk factor for PLN and PALN involvement. These observations support the hypothesis that these cancers metastasize through different pathways and represent distinct clinical entities. According to these observations, it seems appropriate to determine the strategy for lymph node dissection in cases of ovarian cancer according to the type of primary tumour. Type II disease, especially tumour stage > T1, should be treated with lymph node dissection as much as possible. Within each type, there was no difference between PLN and PALN involvement, indicating that there was no pattern in the location of nodal disease.

To explore the important role of the two-tier system in behaviour and biology, the other important issue is to determine whether the risk factors are similar for the two tumour subtypes. The most noteworthy finding of this study was that the risk factors vary according to the type. In other gynaecologic tumours, age and tumour diameter are significant risk factors [21, 35]. However, the roles of age and tumour size in type I were not always the same as those in type II. For PLN involvement in type I cases, beyond advanced tumour stage, there were no other risk factors. However, in the type II group, G3 enhanced the risk, and tumour size > 20 cm reduced the risk. In PALN involvement, G3 also enhanced the risk, but tumour size was not a significant and independent risk factor for node positivity in either the type I or type II group. For PLN + PLAN status, age ≥ 50 y and tumour size > 10- ≤ 15 cm reduced the risk of lymph node metastasis in the type II group. However, they were not significant and independent risk factors for type I. Based on these findings, there is no reason to believe that older patients or patients with larger tumours have a tendency towards lymph node metastasis.

### Survival rates of PLN and PALN metastasis in type I and type II OC

For the entire cohort, the patients with PALN involvement had a significantly more favourable prognostic impact (CSS/OS) than those with PLN and PLN + PALN involvement. However, we should consider that there are different types of ovarian cancer. In type I patients, the survival rate of PALN-positive patients is not better than that of PLN-positive patients and is worse when considering the 10-y OS or CSS. Conversely, the patients with PALN involvement compared to those with PLN metastasis had a significantly more favourable prognosis in type II disease. When predicting the effect of positive lymph nodes on survival, we should first consider the two-tier stratification.

## Conclusion

In conclusion, based on the analysis of a large amount of data, it is possible to determine whether to perform lymph node dissection by assessing tumour stage and two-tier classification. It is noteworthy that, in the present series, two-tier stratification could be more reasonable than pathological grading when assessing lymph node status. It can be deduced that pathological grading should be discussed in the same pathological type. Furthermore, the predictive role of positive lymph nodes varied according to different two-tier stratifications. Patients with PLN involvement are inclined to have longer survival than patients with PALN metastasis in type I, in contrast to the results in type II patients. In patients with stage T1 disease, especially in the type I group, the rate of lymph node metastasis was extremely low, and the clinical prognosis was excellent. Therefore, lymph node dissection was not necessary in patients with early stage T1, especially type I. Consequently, a tailored approach should always be borne in mind. One shortcoming of this study is its retrospective design, while its main value is the large number of patients enrolled. Further prospective studies are necessary to validate our findings.
